# Combined Intramedullary-External K-Wire Fixation for Phalangeal Fractures: Description of a Surgical Technique

**DOI:** 10.1055/s-0046-1824571

**Published:** 2026-10-06

**Authors:** Gabriel Ifran Alves, Antônio Lourenço Severo, Marcelo Barretos de Lemos, Osvandré Lech, Felipe Kowaleski, Ivânio Tagliari

**Affiliations:** 1 Instituto de Ortopedia e Traumatologia (IOT), Hospital São Vicente de Paulo (HSVP), Passo Fundo, RS, Brazil

**Keywords:** external fixators, finger phalanges, fracture fixation, intramedullary, fractures, bone, Kirschner wires, falanges dos dedos da mão, fios de Kirschner, fixação intramedular de fraturas, fixadores externos, fraturas ósseas

## Abstract

Although early mobilization is essential for functional recovery after phalangeal fractures, many fixation methods still require prolonged immobilization, increasing the risk of joint stiffness. We herein describe a simple, safe, and low-cost technique that combines intramedullary Kirschner wires (K-wires) with external connection, adapted from the Ulson method, which was originally developed for distal radius fractures. The procedure consists of closed reduction under fluoroscopy followed by the insertion of two 1.0-mm intramedullary wires. The external ends of these wires are bent and connected, forming a stable frame that enables immediate mobilization without the need for immobilization. The technique provided stable fixation, reliable bone healing, and early rehabilitation, with a low complication rate. It represents a practical and reproducible option, especially useful in resource-limited settings.

## Resumo

A mobilização precoce é fundamental para a recuperação funcional após fraturas das falanges, mas muitos métodos de fixação ainda exigem imobilização prolongada, o que aumenta o risco de rigidez articular. Descrevemos uma técnica simples, segura e de baixo custo, que combina fios de Kirschner intramedulares e ligação externa, adaptada do método de Ulson, originalmente desenvolvido para fraturas do rádio distal. O procedimento consiste em redução fechada sob fluoroscopia, seguida da introdução de 2 fios intramedulares de 1,0 mm, cujas extremidades externas são dobradas e conectadas, e formam um quadro estável que permite mobilização imediata sem necessidade de imobilização. A técnica proporcionou fixação estável, consolidação óssea confiável e reabilitação precoce, com baixa taxa de complicações. Trata-se de uma opção prática e reprodutível, especialmente útil em contextos com recursos limitados.

## Introduction

Phalangeal fractures of the hand are common injuries that require precise management to restore function and prevent stiffness. Stable fractures often respond well to conservative treatment, whereas unstable or displaced fractures require surgical stabilization. Traditional methods, such as Kirschner wires (K-wires), screws, and miniplates, have limitations related to prolonged immobilization, greater soft tissue dissection, or higher costs in resource-limited settings. 1 2 To overcome these limitations, we herein describe a simple and accessible technique that combines intramedullary and external fixations with K-wires, providing stable alignment and enabling early mobilization in patients with fractures of the proximal and middle phalanges. 3 4

## Surgical Technique

The technique is indicated for extra-articular diaphyseal or metaphyseal fractures of the proximal and middle phalanges that require surgical stabilization, in which minimal soft-tissue dissection and early mobilization are desired. 5 6

The procedure may be performed under regional or general anesthesia, with the patient in the supine position and the upper limb supported on an auxiliary table. Closed reduction is achieved under fluoroscopic guidance, applying gentle traction and digital manipulation. Two 1.0-mm K-wires are inserted intramedullarily from medial and lateral entry points at the base of the phalanx, crossing the fracture line and maintaining parallel alignment. Depending on the fracture pattern, the wires may also be inserted retrogradely. The external ends are bent to form a rectangular structure, and they are connected with microporous tape, providing additional stability. Reduction and fixation are confirmed by fluoroscopy, and only a simple dressing is applied, without the need for immobilization ( Figs. 1 2 3 4 ). This construct is based on the Ulson method, which was originally described for distal radius fractures and adapted here for finger fractures. Our technique enables K-wire insertion in a parallel configuration or crossing within the diaphysis, depending on the fracture pattern and bone size. In both configurations, the biomechanical principle remains the same, functioning as an elastic system that generates centripetal compression at the fracture site. Simple dressings are used for postoperative comfort.

**Fig. 1:**
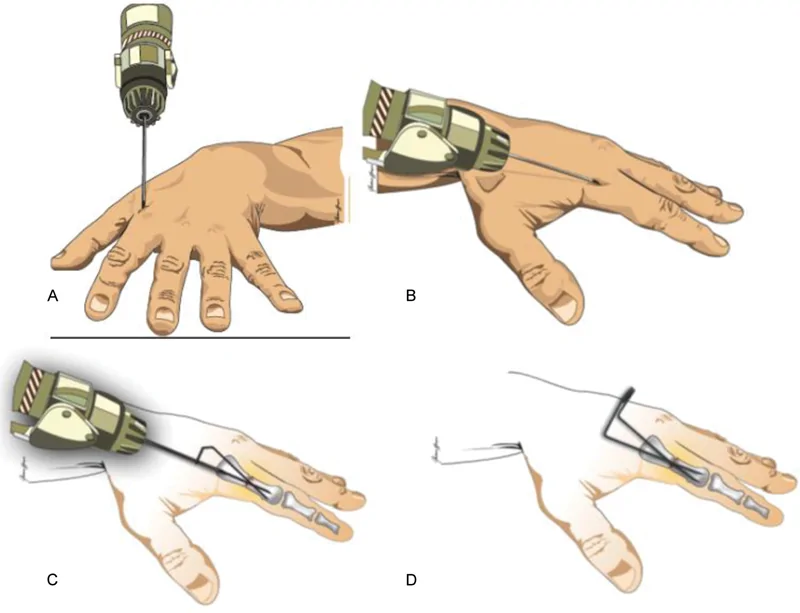
Frontal ( **A** ) and lateral ( **B** ) views of wire insertion; ( **C** ) fracture reduction and fixation; ( **D** ) final “collar-like” construct, stabilized to enable early active mobilization.

**Fig. 2:**
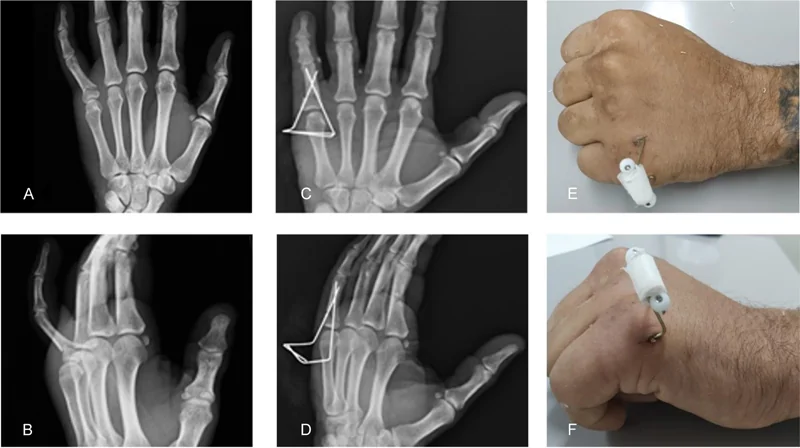
Illustrative case. ( **A,B** ) Fracture of the base of the proximal phalanx of the fifth finger with dorsal displacement; ( **C,D** ) immediate postoperative radiographs; ( **E,F** ) external appearance, demonstrating the patient's ability to actively mobilize the finger. In this case, a small spherical plastic protector was temporarily placed on the external ends of the Kirschner wires for skin protection. This component does not contribute to the stability of the construct and is not routinely used.

**Fig. 3:**
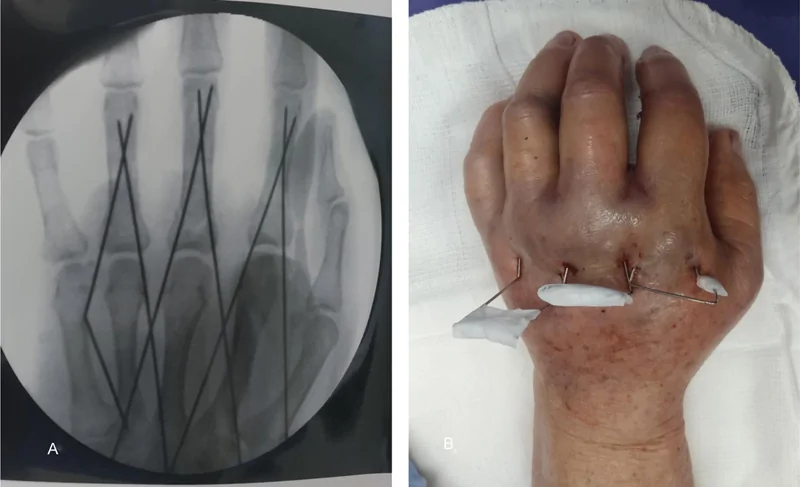
( **A,B** ) Patient with fractures of the proximal phalanges of the second to fourth fingers treated with the combined internal-external fixation method, illustrating the implant configuration in the immediate postoperative period. Prereduction and final follow-up radiographs for this case were not available.

**Fig. 4:**
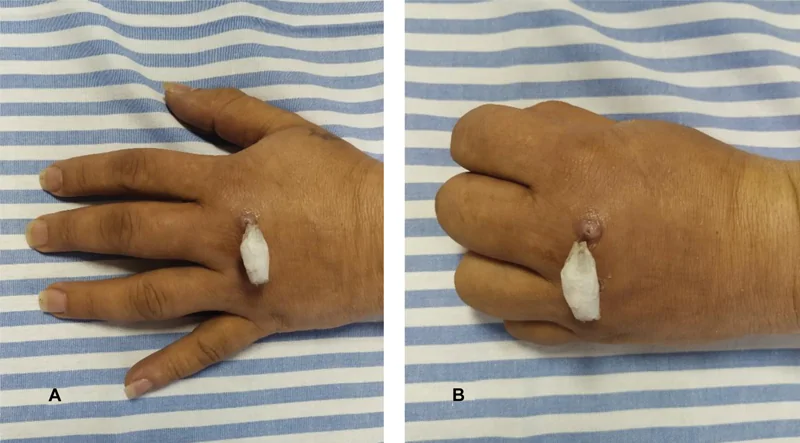
Fingers in ( **A** ) extension and ( **B** ) flexion, clinically demonstrating active mobility in a patient with a proximal phalanx fracture of the middle finger treated with the method herein described. The images are illustrative and intended only to demonstrate the active mobilization enabled by the technique.

Active and passive finger mobilization begin immediately after surgery. Serial radiographs are obtained every 2 weeks to monitor bone healing. The K-wires are removed after radiographic confirmation of bone healing, usually between the fifth and sixth postoperative weeks. After wire removal, the patient continues range-of-motion exercises until complete recovery.

## Outcome

This technique was applied in a consecutive series of 18 patients with proximal and middle phalangeal fractures, most frequently involving the little and ring fingers. Fifteen patients completed the clinical follow-up. All fractures achieved bone healing between 5 and 6 weeks, with early recovery of mobility and a low complication rate. Three cases of superficial pin tract infection were managed conservatively, without loss of reduction, need for hardware removal, or delayed healing. 7

Combined intramedullary-external fixation provides greater rotational stability while preserving the simplicity and minimally-invasive nature of percutaneous fixation. The external connection of the wires enables early mobilization without the need for immobilization, which helps reduce joint stiffness and lowers the risk of tendon adhesions. Moreover, the method is low-cost and easily reproducible, supporting its routine application in different care settings. 8 9 A specific aspect of this configuration is the generation of centripetal forces that promote compression at the fracture site. 10 In the current study, the technique was applied to extra-articular diaphyseal and metaphyseal fractures of the phalanges.

Despite the favorable outcomes, strict fluoroscopic control and precise wire positioning are essential to avoid intra-articular penetration. Expanded clinical experience will better define the indications and long-term outcomes of this technique.

## Conclusion

Combined internal-external fixation with K-wires represents a safe, reproducible, and low-cost option to treat unstable proximal and middle phalangeal fractures. It enables immediate active mobilization and reliable bone healing, with a low complication rate. The method was conceptually adapted from the Ulson technique, which was originally developed for distal radius fractures—an important Brazilian contribution to orthopedic surgery—and its successful application in phalangeal fractures reinforces the potential for adapting established techniques to new anatomical and clinical contexts.
